# Editorial: Microbial Communities of Polar and Alpine Soils

**DOI:** 10.3389/fmicb.2021.713067

**Published:** 2021-09-23

**Authors:** Laura Zucconi, Pietro Buzzini

**Affiliations:** ^1^Department of Ecological and Biological Sciences, University of Tuscia, Viterbo, Italy; ^2^Department of Agricultural, Food and Environmental Sciences - Industrial Yeasts Collection DBVPG, University of Perugia, Perugia, Italy

**Keywords:** soil microorganisms, polar regions, Alpine environments, climate change, microbial communities

## The Three Poles and the Challenges of Climate Change

In recent years Arctic, Antarctic, and Alpine regions have experienced the highest rates of warming worldwide (Zemp et al., 2006; Anisimov et al., 2007). In Arctic and Alpine environments these phenomena are resulting in an increase of the duration of ice-free periods and an overall greening of terrestrial areas. The effects of warming on microbial decomposition of vast carbon pools in permafrost soils have the potential to cause a significant positive feedback to global climate change (Cavicchioli et al., 2019). Climate change in Antarctica, is firstly feared to result in the loss of unique and highly adapted ecosystems, mainly because of shifts in temperature and precipitation regimes, as well as longer term changes in edaphic profiles and the invasion of allochthonous, more competitive species (Convey and Peck, 2019).

Soil microorganisms play a crucial role in mediating carbon and nitrogen balance and other biogeochemical cycles of global importance. Therefore, understanding the soil microbial diversity and ecology, including the ecological drivers that shape microbial communities, may be a key for understanding how biogeochemical cycles will respond to large-scale environmental and climatic changes. Given the key role of microorganisms in maintaining the balance of these environments, they could be viewed both as sentinels and amplifiers of global change (Maloy et al., 2016).

In this framework, the e-book *Microbial Communities of Polar and Alpine Soils* aimed to collect original and noticeable research papers about diversity and functionality of soil microbial communities, their interactions with the other biotic components, including the aboveground plant coverage, and the abiotic factors determinant for the colonization of these habitats, as well their adaptation and resilience abilities to stressing conditions and environmental changes.

## Cold-Living Microorganisms and their Role in Polar and Alpine Environments

This brief editorial summarizes and highlights experimental researches carried out in different environments, ranging from the Qinghai-Tibetan Plateau, to the Arctic, to maritime and continental Antarctica, or spreading across the poles. Different types of soils were studied, from oligotrophic to nitrogen rich soils, from soils underneath plants to thawed permafrost, dry soils, moraines, gullies, polygon soils, or soils around rocks. Some papers dealt with fungi, others with bacteria, others to actinomycetes and/or other organisms.

Ray et al. wanted to define the spread of the “atmospheric chemosynthesis,” a microbial carbon fixation process supporting primary production in dry and nutrient-poor environments. Those genes associated to this process were reported as widespread across cold desert soils, spanning the Tibetan Plateau, and both Antarctic and high Arctic sites.

Otherwise, in some coastal sites of maritime Antarctica, seabirds and marine mammal colonies exert, through the accumulation of their feces and urine, a strong influence on the edaphic N content. Nitrogen cycle in Antarctic tundra ecosystems has also been investigated by Dai et al. and Acuña-Rodríguez et al. The former recorded differences in the denitrification rates and the denitrifier community structures between nitrogen rich soils and animal-lacking tundra soils. The latter observed as fungal symbionts (root endophytes), associated to the only two Antarctic vascular plants *Colobanthus quitensis* and *Deschampsia antarctica*, actively participate in the plants N-uptake, even in non-N limited soils, with positive impacts on plant biomass.

Two contributions by Newsham et al. dealt with the effects of climate change on a single fungal species and the whole communities of soils from maritime Antarctica, respectively. An undescribed member of the order Helotiales was recorded to be superabundant in Antarctic Islands soils under *D. antarctica* (Newsham, Cox et al.). A range of its physiological and morphological features were reported, and an increase of its growth rate was suggested with the rising temperatures that are expected to occur in maritime Antarctica at the end of this century, with the potential loss of ancient C from soils.

Three fungal guilds and growth forms—lichenized and saprotrophic fungi and yeasts—of barren fellfield soils sampled from along a transect encompassing almost the entire maritime Antarctica were studied, in order to define the main environmental factors affecting their richness, relative abundance and taxonomic structure. Air temperature and edaphic factors were reported as main drivers, and discussed in view of the expected future climate changes of the region (Newsham, Davey et al.).

Glacier retreats expose new ice-free barren soils. Some areas have been only recently deglaciated, while others have been mostly deglaciated for millennia. The bacterial communities of these soils have been studied by Almela et al. Those of older soils seemed to be significantly different along the soil profiles, while they were similar in recently (from decades) deglaciated soils. A high degree of heterogeneity was also observed among microbial communities of soils at different sampling locations.

Water tracks, that seasonally flow on the ice-free soils of the McMurdo Dry Valleys in continental Antarctica, are expected to increase with ongoing climate change. They select for bacterial taxa able to cope with challenging environmental conditions. Significant differences in microbial community assembly between on- and off-water track samples, and across two distinct locations were recorded, mainly driven by soil salinity (George et al.). Heterogeneous microbial communities have been found to characterize four different habitats present in higher elevations of Taylor Valley, where biological soil crusts were reported in a gully and moraine next to Canada Glacier, accounted as islands of biodiversity, able to spread organisms and nutrients in the surrounding landscape (Solon et al.).

The Northern high latitudes are a preferential open-air laboratory to study the impact of climate change on soil microbial communities, as they are warming twice as fast as the global average. Four out of 13 contributing articles of the Topic report studies carried out in the Arctic.

Arctic permafrost has become particularly vulnerable to thaw, with consequences on microbial communities that are not yet perfectly known. The bacterial community assembly during permafrost thaw was studied using *in situ* observations and a laboratory incubation of soils in sub-Arctic Sweden, where permafrost thaw has occurred over the past decade. It showed to be driven by randomness (i.e., stochastic processes) immediately after thaw, while environmentally driven (i.e., deterministic) processes became increasingly important in structuring them in post-thaw successions (Doherty et al.). Geospatial differences in hydrology in polygon soils causing gradients in biogeochemistry, soil C storage potential, and thermal properties influencing the distribution of microbial CO_2_ and CH_4_ release, has been studied by Roy Chowdhury et al. by laboratory incubation at increasing temperatures of frozen soil cores collected in the Arctic coastal tundra in Alaska.

A comparison between the microbial community structure of rocks (and surrounding soils) in a high Arctic polar desert (Svalbard) showed significant differences between these substrates. Differences were also reported between rock sandstones and limestones, due to the determinant role of rock physiochemical properties in determining the successful establishment of lichens in lithic environments (Choe et al.).

Shifts in vegetation and soil fungal communities have been recorded in the Arctic tundra as a response to warming temperatures. In this context, fungal community composition in long-term experimental plots simulating the expected increase in summer warming and winter snow depth, were compared using DNA metabarcoding data, and dry and moist tundra appeared to have different trajectories in response to climate change (Geml et al.).

Cultivable actinomycetes isolated from soils near roots of different plants from the Qinghai-Tibetan Plateau were investigated for their enzymatic activity, and for the production of diffusible pigments and organic acids (Ma et al.).

This volume brings together the scientific community to cover all aspects of cold-adapted microorganisms and their role in Polar and Alpine environments. It gives a significant contribution to the long-standing debate on the multiple ecological roles of microorganisms in cold soil ecosystems. We hope that this range of articles will be highly attractive to worldwide researchers involved in the study of soil microbial communities.

## Author Contributions

The authors have equally contributed to the Editorial, and approved it for publication.

## Conflict of Interest

The authors declare that the research was conducted in the absence of any commercial or financial relationships that could be construed as a potential conflict of interest.

## Publisher's Note

All claims expressed in this article are solely those of the authors and do not necessarily represent those of their affiliated organizations, or those of the publisher, the editors and the reviewers. Any product that may be evaluated in this article, or claim that may be made by its manufacturer, is not guaranteed or endorsed by the publisher.

## References

[B1] Anisimov O. A. Vaughan D. G. Callaghan T. V. Furgal C. Marchant H. Prowse T. D.. (2007). Polar regions (arctic and antarctic), in Climate Change 2007: Impacts, Adaptation and Vulnerability. Contribution of Working Group II to the Fourth Assessment Report of the Intergovernmental Panel on Climate Change, eds Parry M. L. Canziani O. F. Palutikof J. P. van der Linden P. J. Hanson C. E. (Cambridge: Cambridge University Press), 653–685. Available online at: https://www.ipcc.ch/site/assets/uploads/2018/02/ar4-wg2-chapter15-1.pdf

[B2] Cavicchioli R. Ripple W. J. Timmis K. N. Azam F. Bakken L. R. Baylis M.. (2019). Scientists' warning to humanity: microorganisms and climate change. Nat. Rev. Microbiol. 17, 569–586. 10.1038/s41579-019-0222-5 31213707 PMC7136171

[B3] Convey P. Peck L. S. (2019). Antarctic environmental change and biological responses. Sci. Adv. 5:eaaz0888. 10.1126/sciadv.aaz0888 31807713 PMC6881164

[B4] Maloy S. Moran M. A. Mulholland M. R. Sosik H. M. Spear J. R. (2016). FAQ: Microbes and Climate Change: Report on an American Academy of Microbiology and American Geophysical Union Colloquium held in Washington, DC, in March *2016* (Washington, DC). 30063309

[B5] Zemp M. Haeberli W. Hoelzle M. Paul F. (2006). Alpine glaciers to disappear within decades? Geophys. Res. Lett. 33:L13504. 10.1029/2006GL026319

